# Copy Number Variations in Candidate Genes and Intergenic Regions Affect Body Mass Index and Abdominal Obesity in Mexican Children

**DOI:** 10.1155/2017/2432957

**Published:** 2017-03-27

**Authors:** Diana Lizzete Antúnez-Ortiz, Eugenia Flores-Alfaro, Ana Isabel Burguete-García, Amélie Bonnefond, Jesús Peralta-Romero, Philippe Froguel, Mónica Espinoza-Rojo, Miguel Cruz

**Affiliations:** ^1^Laboratorio de Investigación en Epidemiología Clínica y Molecular, Facultad de Ciencias Químico Biológicas, Universidad Autónoma de Guerrero, 39089 Chilpancingo, GRO, Mexico; ^2^Unidad de Investigación Médica en Bioquímica, Hospital de Especialidades “Bernardo Sepúlveda”, Centro Médico Nacional Siglo XXI, Instituto Mexicano del Seguro Social, 06725 Ciudad de México, Mexico; ^3^Centro de Investigación sobre Enfermedades Infecciosas, Instituto Nacional de Salud Pública, 62100 Cuernavaca, MOR, Mexico; ^4^CNRS-UMR8199, Lille Pasteur Institute, Lille, France; ^5^Lille University, Lille, France; ^6^European Genomic Institute for Diabetes (EGID), 3508 Lille, France; ^7^Department of Genomics of Common Disease, School of Public Health, Imperial College London, Hammersmith Hospital, London, UK; ^8^Laboratorio de Genómica y Biología Molecular, Facultad de Ciencias Químico Biológicas, Universidad Autónoma de Guerrero, 39089 Chilpancingo, GRO, Mexico

## Abstract

*Introduction*. Increase in body weight is a gradual process that usually begins in childhood and in adolescence as a result of multiple interactions among environmental and genetic factors. This study aimed to analyze the relationship between copy number variants (CNVs) in five genes and four intergenic regions with obesity in Mexican children.* Methods*. We studied 1423 children aged 6–12 years. Anthropometric measurements and blood levels of biochemical parameters were obtained. Identification of CNVs was performed by real-time PCR. The effect of CNVs on obesity or body composition was assessed using regression models adjusted for age, gender, and family history of obesity.* Results*. Gains in copy numbers of* LEPR* and* NEGR1* were associated with decreased body mass index (BMI), waist circumference (WC), and risk of abdominal obesity, whereas gain in* ARHGEF4* and* CPXCR1* and the intergenic regions 12q15c, 15q21.1a, and 22q11.21d and losses in* INS* were associated with increased BMI and WC.* Conclusion*. Our results indicate a possible contribution of CNVs in* LEPR*,* NEGR1*,* ARHGEF4*, and* CPXCR1* and the intergenic regions 12q15c, 15q21.1a, and 22q11.21d to the development of obesity, particularly abdominal obesity in Mexican children.

## 1. Introduction

Excessive body weight is now recognized as an important public health problem worldwide. Obesity is associated with a variety of somatic and psychosocial comorbidities including severe metabolic and cardiovascular complications that normally occur in adulthood. Obesity pathophysiologically induces a metabolically altered state in the majority of obese children, causing dyslipidemia, hypertension, steatosis, and impaired glucose metabolism [1]. In Mexico, based on the latest national health and nutrition survey (ENSANUT 2012), the combined prevalence of overweight and obese school-age children (5–11 years) was 34.4% (19.8% overweight and 14.6% obese) [2]. Increase in body weight is a gradual process that usually begins in childhood and adolescence as a result of multiple interactions among environmental and genetic factors.

In a meta-analysis conducted by a group of researchers around the world, 97 loci (SNPs) were identified to be associated with body mass index (BMI), a measure generally used to define obesity and assess adiposity. These loci account for 2.7% of the variation in BMI and suggest that as much as 21% of BMI variation can be accounted for by common genetic variation. The researchers who performed this analysis provide evidence for particular genes and pathways that affect BMI, including synaptic plasticity and glutamate receptor activity pathways responding to changes in feeding and fasting [3]. An extensive North American, Australian, and European collaborative genome-wide meta-analysis on children of European ancestry uncovered two new obesity loci that have the strongest evidence for association with elevated adiposity in the first 18 years of life, which overlap to a large extent in children as well as in adults [4]. A recent genome-wide study in 47541 children identified three significant loci, which have not been associated with adiposity-related phenotypes previously [5]. However, much is unknown about the variations in the human genome and their relationship with the changes in BMI.

Another type of variation in the structure of the DNA being studied is copy number variants (CNVs), usually defined as genomic segments of size ≥ 1 kb showing copy number variability among individuals in context of a reference genome. CNVs comprise deletions, duplications, insertions, and unbalanced translocations; their presence in the genome comprises approximately 12% of the individual variations and has an effect on gene expression [6]. Despite its importance, the mechanisms of CNV formation and the risk factors involved are poorly understood. As are all mutations, CNVs are heritable and there is a risk of formation of new and deleterious CNVs because of exposure to environmental mutagens that interfere with DNA synthesis and genetic predisposition. At least two distinct pathways are known to be involved in the formation of CNVs-associated diseases: unequal meiotic recombination and replication errors. In particular, CNVs associated with diseases of polygenic origin such as autism [7], metabolic syndrome, obesity [8], and type 2 diabetes (T2D) [9] have been identified.

Association studies of CNVs with obesity often involving extreme obesity phenotypes with or without syndromic features have identified candidate regions near the* NEGR1* locus and chromosome 10q11.22 [10], as well as on chromosomes 11q11 [11] and 10q26.3 [12], among others. A region within chromosome 16p11.2 is particularly well studied, deletions of which are associated with obesity and duplications are associated with an underweight phenotype [13]. Recently, our group analyzed* AMY1* in Mexican children, and the results showed a high copy number of the gene only in children with normal weight. This gene produces salivary amylase and results in improved lipid and carbohydrate metabolism. These variations might contribute to improved carbohydrate metabolism, particularly in the Mexican diet where carbohydrate-rich meals are consumed [14]. Therefore, it is necessary to study the other genes involved in the pathophysiology of obesity in children. The purpose of this study was to evaluate the association between CNVs in specific regions of* LEPR*,* NEGR1*,* ARHGEF4*,* CPXCR1*, and* INS* and in four intergenic regions (1p36.33b, 12q15c, 15q21.1a, and 22q11.21d) and obesity in Mexican children. The studied regions were selected considering loci that were reported or tended to be associated with obesity, especially for those obesity-related alleles reported more than once or those that might affect the genes involved in obesity or related metabolic diseases.

## 2. Materials and Methods

### 2.1. Study Participants

We studied 1423 Mexican children aged 6–12 years, who were residents of Mexico City, not genetically related, did not have any chronic or infectious diseases at the time of the study, and were not under medical treatment. The program moderators provided a detailed explanation of the nature and purpose of the study to participants. The children who agreed to participate in the study, their parents, or legal representatives provided an informed consent. The study protocol was approved by the National Committee and the Ethics Committee of the Mexican Social Security Institute (IMSS) and was conducted in compliance with the Declaration of Helsinki. For each of the participants, anthropometric indicators were determined and questionnaires for family history of metabolic diseases such as obesity and type 2 diabetes were filled out. Weight was measured on a digital scale (Seca, Hamburg, Germany), height was measured with a portable stadiometer (Seca 213, Hamburg, Germany), and waist circumference (WC) was measured just above the uppermost lateral border of the right ilium, at the end of a normal expiration with an anthropometric tape (Seca 201, Hamburg, Germany). Blood samples were obtained after 8 hours of fasting for biochemical measurements and DNA extraction.

### 2.2. Classification of Body Mass Index and Abdominal Obesity

BMI was calculated and classified based on the reference tables for ages 2–20 years provided by the Center for Disease Control and Prevention, 2000 [15]. Normal weight was defined as a BMI-for-age below the 85th percentile, overweight as 85th to 95th percentiles, and obesity as a BMI-for-age above the 95th percentile. Abdominal obesity was defined using reference tables for Mexican-American children when WC was greater than the 75th percentile according to age and gender [16].

### 2.3. Biochemical Measurements

Blood samples were obtained to determine the levels of serum glucose, total cholesterol, triglycerides, LDL-cholesterol, and HDL-cholesterol, using standard methods for the clinical chemistry system ILab 350 (Instrumentation Laboratory SpA, Spain). Adiponectin and leptin concentrations were measured by ELISA (R&D Systems, Minneapolis, MN, USA). Insulin levels were measured using a chemiluminescence method (Inmunolite, France). Insulin resistance was determined with the homeostasis model for insulin resistance (HOMA-IR): [(fasting glucose, mg/dL *∗* Insulin *μ*U/ml)/405] with a value ≥ 3.3, which corresponds to the 90th percentile of the HOMA-IR index.

### 2.4. DNA Extraction and CNV Analysis

Genomic DNA extraction from peripheral blood was performed using columns with a silica membrane (QIAamp DNA Blood Midi/Kit, Qiagen, Germany). To determine the CNVs of genes or intergenic regions, we performed real-time PCR with a specific TaqMan assay and the* RNase P* human TaqMan copy number reference [17–25] (Table 1). We selected genes that had been associated with obesity-related phenotypes before; in these we selected regions that are reported in the database of genomic variations (DGV). These regions were used as an approximation of the copy number in the whole gene. The PCR reaction was performed in the 7900HT Fast Real-Time System (Applied Biosystems, Foster City, CA, USA); as quality control, all samples were analyzed in duplicate. Results were analyzed by the CopyCaller Software v.2.0 (Applied Biosystems, Foster City, CA, USA) to measure the relative copy number of each genome segment in each sample.

### 2.5. Statistical Analysis

We described the sociodemographic and clinical characteristics and the risk factors, expressed in frequencies for qualitative variables and in medians and interquartile range for quantitative variables. For comparison of frequency or medians between groups, the chi square test or Kruskal Wallis test were used, respectively. To determine the association of BMI, WC, or insulin serum levels with the CNVs studied, ANOVA, correlation analysis, and linear regression models were evaluated, considering an alpha of 0.02 after correction by Bonferroni's method. To define the risk of abdominal obesity, logistic regression models were evaluated. Statistical analysis was performed using STATA software v.11.2.

## 3. Results

Based on the percentiles of BMI according to age and gender, all the children were classified into three groups: normal weight, overweight, and obese. The prevalence of normal weight was 50.5%, whereas 21.6% were overweight and 27.9% present were obese. The increase in body weight correlated with low levels of HDL-cholesterol and adiponectin and increased WC and blood pressure were associated with increased levels of glucose, triglycerides, total cholesterol, LDL-cholesterol, leptin, and insulin, compared with these in normal weight children. In addition, children with obesity more frequently presented abdominal obesity, acanthosis nigricans, insulin resistance (IR), and a history of parents or relatives with T2D and/or obesity (Table 2).

We found a negative correlation between BMI and adiponectin levels, as well as between adiponectin and leptin, and adiponectin and insulin, and a positive correlation between BMI with leptin and insulin levels, as well as between the levels of leptin and insulin (Table 3).

**Figure 1:**
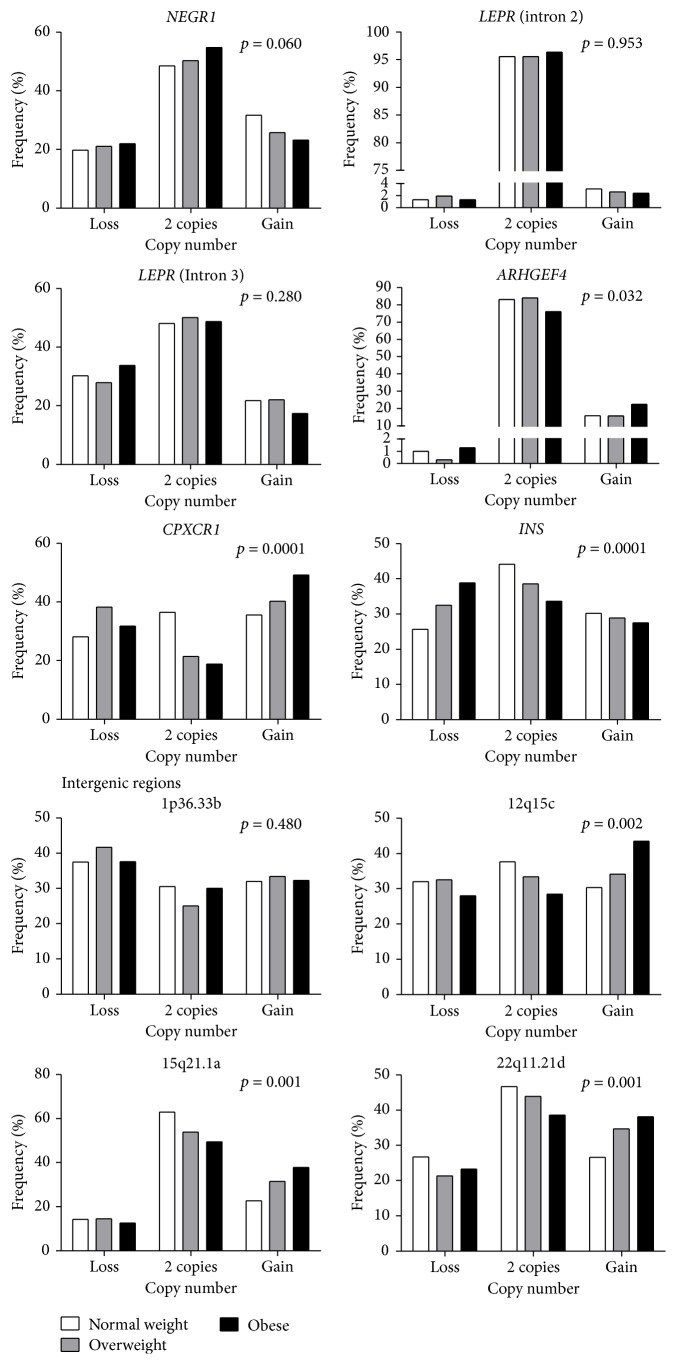
Frequency of copy number in studied regions by BMI group. The *p* value was calculated using the *χ*^2^ test (Table S1).

**Figure 2:**
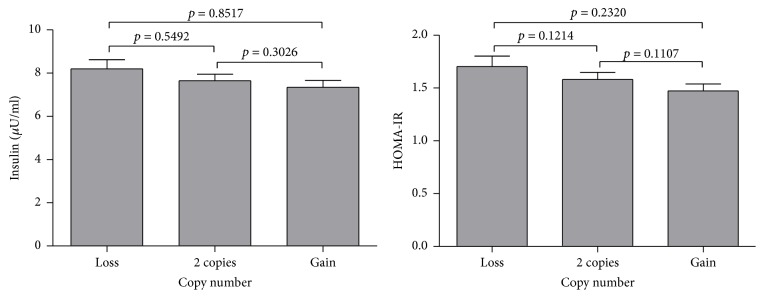
Relationship between insulin levels and insulin resistance with CNVs in the* INS* gene. Variance analysis (ANOVA) was performed to determine the relationship between levels of insulin or HOMA-IR index and CNVs. Pair comparisons by test of Bonferroni.

The analysis of CNVs in six regions corresponded to five genes previously associated with obesity,* LEPR* (intron 2 and intron 3),* NEGR1*,* ARHGEF4*,* CPXCR1*, and* INS*, and four intergenic regions (1p36.33b, 12q15c, 15q21.1a, and 22q11.21d). For all these CNVs, children more frequently presented 2 copies. We classified the CNVs in three categories: loss (0 or 1 copies), reference group (2 copies), and gain (3 or more copies). We found significant differences in CNV frequency between children with obesity compared with that in normal weight children for 3 of the 6 intragenic regions and 3 of the 4 intergenic regions tested. For* ARHGEF4*, we found that children with obesity more frequently showed gains (22.6%) as also observed for* CPXCR1* (49.2%), whereas for* INS* losses occurred most frequently in obese children (25.7%). Regarding intergenic regions, it was found that, in children with obesity, gains occurred more frequently at the positions 12q15c (43.5%), 15q21.1a (37.9%), and 22q11.21d (38.1%) compared with those in children of normal weight (Figure 1, Table S1 in Supplementary Material available online at https://doi.org/10.1155/2017/2432957). Furthermore, the relationship between serum insulin levels and insulin resistance with CNV was assessed in* INS*. Insulin levels and HOMA-IR index were not significantly different from each other in the three types of CNVs (Figure 2). No significant associations were found between serum levels of total cholesterol, HDL-c, or LDL-c with the CNVs in the genes or intergenic regions analyzed.

**Figure 3:**
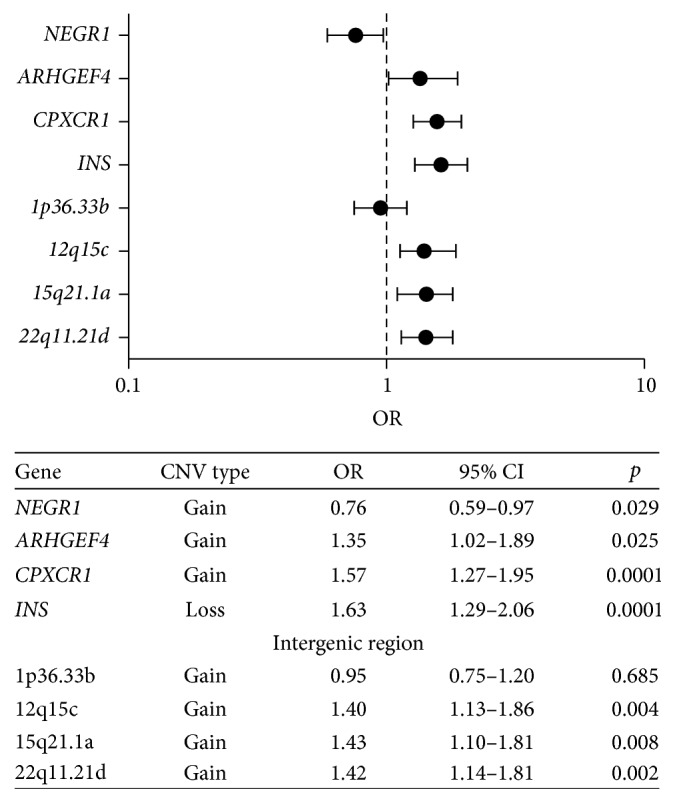
Association between the CNVs and abdominal obesity in Mexican children. The forest plot shows the results of the logistic regression models adjusted for age and gender; for the* ARHGEF4*,* NEGR1,* and* CPXCR1* and intergenic regions the reference group was defined as individuals with loss copy number (0–2) and compared with the group with the highest copy number (≥3), while for the* INS* the reference group was of children with the highest copy number (≥2) and compared with the group with loss copy number (0-1).

We observed that children with gains in intron 3 of* LEPR* showed a decrease in WC, as well as in the case of* NEGR1*, which correlates with a significant decrease in BMI and WC. On the other hand, children with gains in* CPXCR1* and* ARHGEF4* showed a significant increase in BMI. In the case of* INS*, it was observed that children with losses had a significantly greater BMI and WC. Analysis of the intergenic regions showed that the gain in the 12q15c region was associated with increased BMI, the 15q21.1a region with an increase in WC, and the 22q11.21d region with increased BMI and WC (Table 4). Children with gains in* NEGR1* had a 24% lower risk of abdominal obesity (OR = 0.76; 95% CI: 0.59–0.97), whereas children with gains in* ARHGEF4* and* CPXCR1* and losses in* INS* presented 1.35 (*p* = 0.025), 1.57 (*p* < 0.001), and 1.63 (*p* < 0.001) times higher risk of abdominal obesity, respectively, compared with that in children with low copy numbers (0–2). We also observed an association between abdominal obesity with the copy number of intergenic regions: 12q15c (OR = 1.40; 95% CI: 1.13–1.86), 15q21.1a (OR = 1.43; 95% CI: 1.10–1.81), and 22q11.21d (OR = 1.42; 95% CI: 1.14–1.81) (Figure 3).

## 4. Discussion

Genetic association studies are useful for understanding the pathogenesis of polygenic diseases such as obesity, allowing us to study the simultaneous participation of several genes and genetic variants [26]. Although at least 97 loci are associated with the risk of obesity, many of the identified genes are not known to have any role in the biology of the disease under study and include described associations with genomic regions whose functions are yet unknown. Currently, the participation of CNVs has been proposed to play a role in disease development and progression [3].

Our data show that circulating adiponectin was decreased in obese children with a strongly negative correlation between serum adiponectin and BMI, whereas serum leptin was increased 3-fold in these children. Leptin resistance appears to be a mechanism contributing to the burden of obesity that extends across multiple organs. Leptin is involved in food intake, body weight, energy expenditure, and neuroendocrine functions. It has also been associated with the percentage of visceral and body fat, BMI, hip circumference, and glucose levels. Several studies have reported that mutations in the genes encoding leptin* (LEP)* and its receptor* (LEPR)* have been associated with hyperphagia and morbid obesity [27, 28]. Jeon et al. show that higher copy numbers of the* LEPR* exon 2 may contribute to higher transcriptional activity of* LEPROT* by a gene dosage effect, which may be accordingly responsible for* LEPR* downregulation in T2D patients. In our study, we did not determine whether the presence of CNVs in introns 2 and 3 of* LEPR* affects its expression; we only observed a difference in the copy number in these regions, suggesting that the region of intron 2 is more resistant to the formation of new CNVs by the proposed mechanisms (the unequal meiotic recombination and replication errors) [29]. Although we suggest that the regulatory regions refer to those that may be contained within the studied intergenic regions and may affect the expression of nearby genes, this does not specifically refer to the* LEPR* gene.

In this study, we found significant differences in the copy numbers among children with obesity compared to those in normal weight children for* LEPR*,* NEGR1 ARHGEF4*,* CPXCR1*, and* INS* and four intergenic regions previously associated with obese children. Additional significant associations with other obesity-related features such as waist circumference and abdominal obesity consistent with the associations with BMI were also found. Jeon et al., in an analysis of adult Koreans, found that CNVs located in the region of exon 2 of* LEPR* contribute to deregulation of* LEPR*, interrupting its signal, and resulting in an association with increased body weight [29]. We found that the CNVs in intron 2 have no relation with BMI or WC, whereas the CNVs in intron 3 contribute to decreased WC, which could influence gene expression depending on the region where it is located.

Furthermore,* NEGR1* located on chromosome 1p31.1 encodes a protein of the immunoglobulin family with widespread expression in the hypothalamus, hippocampus, and cerebellum and is involved in the differentiation and maturation of neurons and adipocytes [30]. Variations in this gene have been associated with obesity and insulin resistance [31]. Furthermore, Willer et al. found that an elimination of 45 kb, which removed the conserved elements upstream of this gene, is associated with increased BMI and delayed development [32]. In our study, an increase in the copy number of this gene was associated with decreased BMI (0.67 kg/m^2^) and WC (2.01 cm); on the other hand, it reduced the risk of abdominal obesity (OR = 0.76).

*ARHGEF4* is located in 2q21.1c and encodes a guanine nucleotide exchanger involved in the signaling by the proteins Rac and Rho, which are involved in several metabolic processes [33]. The CNVs of this gene are associated with delayed development and neural alterations [34], as well as resistance to insulin [35]. We found that an increase in the CNVs of this gene was associated with increased risk of abdominal obesity (OR = 1.35).

*CPXCR1* is located in Xq21.3, and this region has been associated with the development of cleft lip and cleft palate [36], though the mechanism by which this disease develops has not been defined. In terms of CNVs, Rocca et al. found that this region is associated with the phenotypes of the Klinefelter syndrome (KS) [37], which has a wide range of phenotypes. The common characteristics include small testes and infertility, but KS subjects are at increased risk of hypogonadism, cognitive dysfunction, obesity, T2D, osteoporosis, and autoimmune disorders [38]. We found that this region contributed to the risk of developing abdominal obesity (OR = 1.57) and increased WC (2.10 cm) and BMI (0.71 kg/m^2^).

*INS* is located in 11p15.5 and encodes preinsulin that when processed in proteasomes becomes the active form of insulin and maintains glucose and lipid homeostasis. Polymorphisms in this gene have been associated with the development of MODY diabetes [39], whereas the C109Y mutation is associated with the development of neonatal diabetes [40]. In this study, we found that losses in this gene increased the risk of abdominal obesity (OR = 1.63) and were associated with an increase in BMI (0.77 kg/m^2^) and WC (2.20 cm). However, these losses were not associated with serum insulin levels, suggesting that these variations may influence protein functionality but not its expression.

In relation to variations in the intergenic regions, little is known about their biological role in the genome. However, it is possible that these regions contain regulatory elements such as enhancers and repressors that modify the expression of nearby genes. The CNVs may exert their effects on the causative gene whose distance can be as far as 1 Mb away [41]. The regions studied are close to* MDM2* (12q15),* LIPC* (15q21.1), and* MAPK1* (22q11.21).* MDM2* encodes an ubiquitin ligase, which marks tumor suppressor proteins such as p53 for proteasomal degradation; its overexpression has been associated with increased susceptibility to tumor formation [42].* LIPC* encodes a hepatic lipase with hydrolase activity for triglycerides and variants of this gene have been implicated in increased risk of cardiovascular disease [43] and T2D [44].* MAPK1* (also called p38-MAPK) is a member of the serine/threonine kinase, which acts as an essential component of the MAP kinase signal transduction pathway. Depending on the cellular context, the MAPK/ERK cascade mediates diverse biological functions such as cell growth, adhesion, survival, and differentiation through regulation of transcription, translation, and cytoskeletal rearrangements [45]. Involvement of p38-MAPK has been shown in adipogenesis and hyperglycemia in cell lines, whereas, in animal models with a high-fat diet, it has been associated with the development of obesity, hyperglycemia, glucose intolerance, and insulin intolerance [46].

Bae et al. found three new regions with CNVs (chr15: 45994758–45999227, chr22: 20722473–21702142, and chr18: 3559620–3561217) that were significantly associated with the risk of T2D, particularly, the 15q21.1 region of chr15: 45994758–45999227 [9]. We found a significant association between the gain in the copy number in intergenic regions with abdominal obesity and increasing BMI. Our results suggest that the effect of CNVs depends on their location within the genome, which could modify the translation and transcription levels of nearby genes. Thus, CNVs would result in compromised metabolic pathways related to nutrient absorption, leading to the development of obesity in childhood. Experimental studies are needed to confirm or refute this hypothesis.

In this study, 1423 children were included, which allowed the detection of low frequency CNVs, thus strengthening the impact of the study. However, there were a few limitations as well. We only demonstrated the presence of CNVs related to obesity in Mexican children; however, the pathogenic mechanisms by which these CNVs can influence the development of obesity are yet to be determined. The associations observed between CNVs and obesity are specific to our population, and so the knowledge of their genetic structure is of fundamental importance in characterizing the associated genetic variants in greater detail. It should be considered that the frequencies of these genetic variants differ from those in the population where they were originally identified. However, it is necessary to establish suitable designs that take into account the appropriate inclusion criteria for cases and controls, homogeneity, and appropriate sample size based on the prevalence of obesity. Finally, it is necessary to repeat this study in other populations to validate our findings.

## 5. Conclusions

Our study provides evidence of regions with copy number variations related to obesity, principally abdominal obesity, and its effect on BMI in Mexican children, which are important genetic markers. It is important to continue searching for these variations and determine their pathogenic mechanisms to prevent and control obesity and to consider the genetic differences due to population ancestry.

## Supplementary Material

Table S1 shows the significant differences in CNV frequency between children with obesity compared with that in normal weight children for 3 of the 6 intragenic regions and 3 of the 4 intergenic regions tested. For ARHGEF4, we found that children with obesity more frequently showed gains (22.6%) as also observed for CPXCR1 (49.2%), whereas for INS losses occurred most frequently in obese children (25.7%). Regarding intergenic regions, it was found that, in children with obesity, gains occurred more frequently at the positions 12q15c (43.5%), 15q21.1a (37.9%), and 22q11.21d (38.1%) compared with those in children of normal weight.

## Figures and Tables

**Table 1:** Copy number variants selected in five genes and four intergenic regions.

Assay ID	DGV	Chromosome position	Gene	Location	Reference
Hs06530590_cn	esv3586400	Chr1: 72516113–72546537	*NEGR1*	Intron 1	[17]
Hs06585085_cn	nsv830081	Chr1: 65886080–66062800	*LEPR*	Intron 2	[18]
Hs06571926_cn	nsv823109	Chr1: 65923256–66024320	*LEPR*	Intron 3	[19]
Hs02877230_cn	dgv4075n100	Chr2: 131477948–132279313	*ARHGEF4*	Exon 1	[18]
Hs02728909_cn	nsv515219	ChrX: 87893400–88151600	*CPXCR1*	Exon 1	[20]
Hs01116764_cn	dgv1566n54	Chr11: 2176852–2181266	*INS*	Exon 2	[21]
Hs03336984_cn	nsv471522	Chr1: 522139–756783	Intergenic	1p36.33b	[22]
Hs06941897_cn	nsv821005	Chr12: 69819095–69819779	Intergenic	12q15c	[23]
Hs05382010_cn	esv3892684	Chr15: 45659188–46372824	Intergenic	15q21.1a	[24]
Hs04082205_cn	nsv834135	Chr22: 20724378–20845563	Intergenic	22q11.21d	[25]

DGV: database of genomic variants. Assay ID: https://www.thermofisher.com/mx/es/home/brands/applied-biosystems.html.

**Table 2:** Anthropometric and clinical characteristics of participating children in the study.

Factor	Normal weight	Overweight	Obese	*p*
	719 (50.5%)	308 (21.6%)	396 (27.9%)	
Gender *n* (%)				
Male	378 (52.6)	144 (46.8)	221 (55.8)	0.107^a^
Female	341 (47.4)	164 (53.3)	175 (44.2)	
Age (years)	9 (7–11)	9 (8–11)	9 (8–11)	0.066^b^
BMI (kg/m^2^)	16.4 (15.2–17.9)	20.3 (18.8–21.7)	24.1 (22–26.8)	<0.001^b^
Waist circumference (cm)	57.6 (53.7–62.9)	68.1 (62.8–73.9)	78 (72.2–86.2)	<0.001^b^
Abdominal obesity, *n* (%)				
No	694 (96.5)	191 (62)	31 (7.8)	<0.001^a^
Yes	35 (3.5)	117 (38)	365 (92.2)	
SBP (mmHg)	95 (90–100)	100 (90–105)	100.5 (95–110)	<0.001^b^
DBP (mmHg)	65 (60–70)	69 (60–70)	70 (60–73)	<0.001^b^
Glucose (mg/dl)	81 (75–87)	82 (77–88)	83 (77–89)	0.007^b^
Cholesterol (mg/dl)	152 (132–175)	158 (137.5–177)	157.5 (136–181.5)	0.008^b^
HDL-c (mg/dl)	54 (45–61)	49.5 (41–57.5)	43 (37–52)	<0.001^b^
LDL-c (mg/dl)	96 (82–114)	104.5 (88–120)	105.5 (89.5–122)	<0.001^b^
Triglycerides (mg/dl)	70 (55–90)	87.5 (66.5–119.5)	103 (76–147.5)	<0.001^b^
Adiponectin (*μ*g/ml)	14.5 (12.5–16.4)	14.1 (12.3–16.5)	13.6 (11.8–16.0)	0.009^b^
Leptin (ng/ml)	10.2 (5.8–17.4)	22.4 (14.8–29.5)	30.9 (22.6–38.4)	<0.001^b^
Insulin (*μ*U/ml)	4.6 (2.6–7.4)	7.4 (4.3–11)	9.7 (4.8–16.2)	<0.001^b^
HOMA-IR, *n* (%)				
<percentile 90	703 (97.7)	289 (93.8)	284 (71.7)	<0.001^a^
≥percentile 90	16 (2.3)	19 (6.2)	112 (28.3)	
Acanthosis, *n* (%)				
No	642 (89.3)	225 (73.1)	148 (37.4)	<0.001^a^
Yes	77 (10.7)	83 (26.9)	248 (62.6)	
T2D family history, *n* (%)				
No	655 (91.1)	268 (87)	340 (85.9)	0.017^a^
Yes	64 (8.9)	40 (13)	56 (14.1)	
Obesity family history, *n* (%)				
No	390 (54.2)	139 (45.1)	137 (34.6)	<0.001^a^
Yes	329 (45.8)	169 (54.9)	259 (65.4)	

Data are reported as medians (25th–75th) or as noted in table. ^a^Chi-square test; ^b^Kruskall Wallis test. BMI: body mass index; SBP: systolic blood pressure; DBP: diastolic blood pressure; HDL-c: high density lipoprotein-cholesterol; LDL-c: low density lipoprotein-cholesterol; HOMA-IR: homeostatic model assessment-insulin resistance; T2D: type 2 diabetes.

**Table 3:** Correlation between serum levels of adiponectin, leptin, and insulin with BMI.

Factors	*r*	*p*
BMI/adiponectin	−0.1287	<0.001
BMI/leptin	0.7112	<0.001
BMI/insulin	0.4693	<0.001
Adiponectin/leptin	−0.0803	0.007
Adiponectin/insulin	−0.1064	<0.001
Leptin/insulin	0.4145	<0.001

*r*: Spearman correlation coefficient. BMI: body mass index.

**Table 4:** Effect of copy number in genes and intergenic regions on body mass index and waist circumference.

	Factor	Type	*β* (95% CI)^a^	*p*
Gene				
*LEPR *(intron 2)	BMI	Gain	−0.26 (−1.51; 0.98)	0.677
	WC		−1.37 (−4.64; 1.91)	0.413
*LEPR *(intron 3)	BMI	Gain	−0.50 (−1.01; 0.02)	0.057
	WC		−1.85 (−3.21; −0.49)	0.008
*NEGR1*	BMI	Gain	−0.64 (−1.10; −0.19)	0.006
	WC		−2.03 (−3.24; −0.83)	0.001
*ARHGEF4*	BMI	Gain	0.63 (0.11; 1.15)	0.017
	WC		1.45 (0.09; 2.81)	0.037
*CPCXR1*	BMI	Gain	0.66 (0.21; 1.11)	0.004
	WC		2.10 (0.89; 3.30)	0.001
*INS*	BMI	Loss	0.77 (0.30; 1.25)	0.002
	WC		2.20 (0.94; 3.45)	0.001

Intergenic regions				
1p36.33b	BMI	Gain	0.06 (−0.46; 0.57)	0.829
	WC		−0.13 (−1.47; 1.21)	0.852
12q15c	BMI	Gain	0.88 (0.36; 1.41)	0.001
	WC		2.04 (0.65; 3.43)	0.004
15q21.1a	BMI	Gain	0.67 (0.20; 1.14)	0.005
	WC		1.98 (0.75; 3.21)	0.002
22q11.21d	BMI	Gain	1.09 (0.59; 1.59)	<0.0001
	WC		2.61 (1.29; 3.92)	<0.0001

^a^Linear regression models adjusted for age, gender, and family history of obesity. Children with 2 copies were chosen as the reference group. BMI: body mass index; WC: waist circumference.
