# Supplementary material for: Copy Number Variations in Candidate Genes and Intergenic Regions Affect Body Mass Index and Abdominal Obesity in Mexican Children
Source: Biomed Res Int. 2017 Mar 27;2017:2432957. doi: 10.1155/2017/2432957 (PMC5385910; doi:10.1155/2017/2432957)
Supplement: Supplementary file 1 — Table S1 shows the significant differences in CNV frequency between children with obesity compared with that in normal weight children for 3 of the 6 intragenic regions and 3 of the 4 intergenic regions tested. For ARHGEF4, we found that children with obesity more frequently showed gains (22.6%) as also observed for CPXCR1 (49.2%), whereas for INS losses occurred most frequently in obese children (25.7%). Regarding intergenic regions, it was found that, in children with obesity, gains occurred more frequently at the positions 12q15c (43.5%), 15q21.1a (37.9%), and 22q11.21d (38.1%) compared with those in children of normal weight. [file 2432957.f1.docx]

TABLE S1. Frequency of CNVs per body mass index group

|  | Type of CNV | Normal weight  719 (50.53%) | Overweight  308 (21.64%) | Obese  396 (27.83) | p^a^ |
| --- | --- | --- | --- | --- | --- |
| Gene |  |  |  |  |  |
| *LEPR* | Loss (0-1)  2 copies  Gain (≥3) | 9 (1.3)  687 (95.5)  22 (3.1) | 6 (1.9)  294 (95.5)  8 (2.6) | 5 (1.3)  381 (96.3)  10 (2.4) | 0.953 |
| *LEPR* | Loss (0-1)  2 copies  Gain (≥3) | 217 (30.2)  345 (48)  157 (21.8) | 86 (27.9)  154 (50)  68 (22.1) | 134 (33.8)  193 (48.7)  69 (17.4) | 0.280 |
| *NEGR1* | Loss (0-1)  2 copies  Gain (≥3) | 142 (19.8)  349 (48.5)  228 (31.7) | 65 (21.1)  155 (50.3)  88 (25.7) | 87 (22)  217 (54.8)  92 (23.2) | 0.060 |
| *ARHGEF4* | Loss (0-1)  2 copies  Gain (≥3) | 7 (1)  595 (83)  115 (16) | 1 (0.3)  255 (83.9)  48 (15.8) | 5 (1.3)  296 (76.1)  88 (22.6) | 0.032 |
| *CPXCR1* | Loss (0-1)  2 copies  Gain (≥3) | 202 (28.1)  262 (36.4)  255 (35.5) | 118 (38.3)  66 (21.4)  124 (40.3) | 126 (31.8)  75 (18.9)  195 (49.2) | 0.0001 |
| *INS* | Loss (0-1)  2 copies  Gain (≥3) | 185 (25.7)  317 (44.1)  217 (30.2) | 100 (32.5)  119 (38.6)  89 (28.9) | 154 (38.9)  133 (33.6)  109 (27.5) | 0.0001 |
| Intergenic regions | | | | | |
| 1p36.33b | Loss (0-1)  2 copies  Gain (≥3) | 270 (37.5)  219 (30.5)  230 (32.0) | 128 (41.6)  77 (25.0)  103 (33.4) | 149 (37.6)  119 (30.1)  128 (32.3) | 0.480 |
| 12q15c | Loss (0-1)  2 copies  Gain (≥3) | 230 (32.0)  271 (37.7)  218 (30.3) | 100 (32.5)  103 (33.4)  105 (34.1) | 111 (28.0)  113 (28.5)  172 (43.5) | 0.002 |
| 15q21.1a | Loss (0-1)  2 copies  Gain (≥3) | 103 (14.3)  452 (62.9)  163 (22.8) | 45 (14.6)  166 (53.9)  97 (31.5) | 50 (12.6)  196 (49.5)  150 (37.9) | 0.001 |
| 22q11.21d | Loss (0-1)  2 copies  Gain (≥3) | 192 (26.7)  336 (46.7)  191 (26.6) | 66 (21.4)  135 (43.9)  107 (34.7) | 92 (23.3)  153 (38.6)  151 (38.1) | 0.001 |

The data indicate n (%). ^a^  Chi-square test.
